# Deep generative models for automated muscle segmentation in computed tomography scanning

**DOI:** 10.1371/journal.pone.0257371

**Published:** 2021-09-10

**Authors:** Daisuke Nishiyama, Hiroshi Iwasaki, Takaya Taniguchi, Daisuke Fukui, Manabu Yamanaka, Teiji Harada, Hiroshi Yamada

**Affiliations:** Department of Orthopedic Surgery, Wakayama Medical University, Wakayama, Japan; Polytechnical Universidad de Madrid, SPAIN

## Abstract

Accurate gluteus medius (GMd) volume evaluation may aid in the analysis of muscular atrophy states and help gain an improved understanding of patient recovery via rehabilitation. However, the segmentation of muscle regions in GMd images for cubic muscle volume assessment is time-consuming and labor-intensive. This study automated GMd-region segmentation from the computed tomography (CT) images of patients diagnosed with hip osteoarthritis using deep learning and evaluated the segmentation accuracy. To this end, 5250 augmented pairs of training data were obtained from five participants, and a conditional generative adversarial network was used to identify the relationships between the image pairs. Using the preserved test datasets, the results of automatic segmentation with the trained deep learning model were compared to those of manual segmentation in terms of the dice similarity coefficient (DSC), volume similarity (VS), and shape similarity (MS). As observed, the average DSC values for automatic and manual segmentations were 0.748 and 0.812, respectively, with a significant difference (p < 0.0001); the average VS values were 0.247 and 0.203, respectively, with no significant difference (p = 0.069); and the average MS values were 1.394 and 1.156, respectively, with no significant difference (p = 0.308). The GMd volumes obtained by automatic and manual segmentation were 246.2 cm^3^ and 282.9 cm^3^, respectively. The noninferiority of the DSC obtained by automatic segmentation was verified against that obtained by manual segmentation. Accordingly, the proposed GAN-based automatic GMd-segmentation technique is confirmed to be noninferior to manual segmentation. Therefore, the findings of this research confirm that the proposed method not only reduces time and effort but also facilitates accurate assessment of the cubic muscle volume.

## Introduction

The gluteus medius (GMd) plays a crucial role in stabilizing the hip joint in many daily activities, and the atrophy of this muscle can decrease the strength of the hip abductor muscle, leading to walking instability on the frontal plane. Trendelenburg gait—in which the pelvis inclines toward the swinging leg owing to the insufficient contraction of the GMd to keep the pelvis horizontal when the leg on the affected side is supporting weight while walking—is a widely known gait pattern. Patients with hip osteoarthritis (OA) experience leg shortening because of lateral cranial subluxation or the flattening of the femur head with progress in deformity, and hip flexion limitation becomes prominent. Consequently, the looseness and dysfunction of the GMd lead to atrophy of the GMd.

Accurate evaluation of the GMd volume may be useful for analysis of the muscular atrophy state because of the disease and understanding of the recovery process by rehabilitation. Many studies [1, 2] have selected several slices of a representative axial, extracted a muscle contour, and used the cross-sectional area (CSA) of the muscle as a substitute for volume evaluation. Uemura et al. [2] measured the CSA of the GMd on the plane perpendicular to the anterior pelvic plane through the bilateral anterior superior iliac spines to track chronological changes. However, when the CSA is used as a substitute for volume evaluation, no rationale is given for the slice to be selected.

In addition, some reports have suggested that cubic muscle volume measurements are required for accurate muscle volume evaluation [3, 4]. Amini et al. [3] showed that the use of the total psoas volume to define sarcopenia is associated with both short- and long-term outcomes following resection of pancreatic cancer. They concluded that it might be more efficient to assess the entire volume of the psoas muscle compared with assessing the total psoas area using a single axial image, to define sarcopenia.

Thus, evaluation using the cubic volume is desirable, but it is still difficult to employ in clinical practice because segmentation of the muscle regions of medical images required for cubic muscle volume analysis requires considerable time and effort [5].

### Contributions

This paper makes the following contributions.

1. A deep learning-based method for automatic segmentation of GMd regions from computed tomography (CT) images of hip OA patients is proposed.
2. Three-dimensional volume evaluation is performed on medical images by segmenting the structure of interest from all slices wherein the structure appears, multiplying the CSA thereof with the tomographic thickness, and defining the result as a volume.
3. Automated muscle segmentation considerably reduces the time and effort required for manual segmentation. Moreover, it facilitates the accurate assessment of the cubic muscle volume, thereby enabling its application in medical practice.
4. Owing to its excellent reproducibility, there is little variation in the segmentation result between procedures; furthermore, this approach is expected to improve the quantification of chronological changes.
5. Segmentation accuracy is evaluated using similarity metrics.

### Literature review

In recent years, deep learning and generative models have been widely adopted in musculoskeletal radiology [6, 7]. Several methods proposed for the automated detection, grading, and localization of abnormalities on spinal sagittal magnetic resonance imaging have yielded performances comparable to those of human examiners [8, 9].

Several studies focused on automatic muscle segmentation from CT images have been conducted. Lee et al. [10] proposed a method for automatically segmenting muscles from CT cross-sectional images at the third lumbar level using deep learning; however, the muscles were treated as a lump and not individual segments. Castiglione et al. [11] proposed a U-Net-based convolutional neural network model that could accurately identify the L3 levels and segment the skeletal muscle in pediatric CT scans. Kamiya et al. [12] proposed an automatic segmentation method for the psoas major muscle using a shape model; however, it is difficult to apply this approach to the GMd because the periphery of the psoas major muscle is covered with adipose tissue, and the boundary is clear. Yokota et al. [13] proposed automated muscle segmentation of the hip joint muscle using the hierarchical multi-atlas method and by employing 3D CT data of patients with hip OA. Their method overcomes the considerable shape variability while simultaneously segmenting the 19 skeletal muscles around the hip and thigh. Hiasa et al. [14] proposed a method using Bayesian convolutional neural networks with U-Net and Monte Carlo dropout for the automatic segmentation of individual muscles from clinical CT images; their results showed significant improvements compared to the hierarchical multi-atlas method.

None of the studies cited above investigated the application of conditional generative adversarial networks (GANs) to muscle segmentation in CT images. GAN is good at generating clean images, and it is more popular than flow-based generative models and variational autoencoders. Because of their simple structure, GANs have been used for training major machine learning frameworks, such as TensorFlow, PyTorch, and Chainer. Unlike GANs, for which several implementations have been prepared, the methods developed in the above-cited studies are difficult to disseminate widely in clinical practice.

## Materials and methods

This study has been approved by the institutional review board of Wakayama Medical University Hospital (No. 2907). In this retrospective study, preoperative hip CT scans of patients with hip OA performed at Wakayama Medical University Hospital between May and September 2017 were reviewed. The inclusion criteria were as follows: 1) the patient planned to undergo elective primary total hip replacement for OA; 2) a complete preoperative hip CT scan was available, including the whole pelvis and knee; and 3) no evidence of hip or hamstring contractures was found. The exclusion criteria were as follows: 1) the patient had a history of surgery for pelvic trauma, infections, or tumors, and 2) the patient had a history of previous hip surgeries for femoral trauma. The contracture of the hamstrings was defined as <70° in the straight leg raise test. Hip contracture was defined as <90° flexion, ≤15° abduction, ≤10° external rotation, or ≥20° flexion contracture. Written comprehensive informed consent regarding the use of medical data in research was obtained from all participants.

Five female and two male participants (14 hips) were investigated in this study. The mean age was 72.4 years (range, 65–82 years). The primary diagnosis was OA in nine hips and prosthetic joint in two hips, and three hips were radiographically normal. The demographic and pathological characteristics of the seven participants are listed in Table 1.

**Table 1:** Demographic and pathological characteristics of the seven participants.

Participant	Dataset	Gender	Age (years)	Right hip	Left hip	Number of slices including GMd
**1**	Training	F	65	Crowe 1	Crowe 1	98
**2**	Training	F	69	Crowe 1	Normal	101
**3**	Training	F	75	Prosthetic joint	Crowe 1	100
**4**	Training	M	66	Crowe 1	Normal	117
**5**	Training	M	71	Crowe 1	Prosthetic joint	109
**6**	Validation	F	82	Normal	Crowe 1	80
**7**	Test	F	79	Crowe 1	Crowe 1	86
		Mean	72.4		Total	691

Crowe 1: Crowe classification type 1 for hip dysplasia; GMd: gluteus medius; Mean: average.

**Fig 1:**
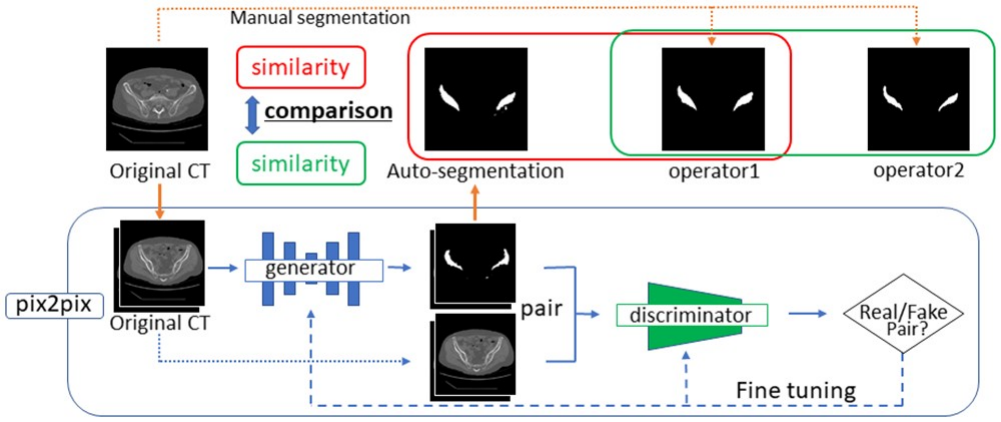
Overview of proposed automatic segmentation system.

All CT scans were obtained using a helical CT scanner (LightSpeed VCT 64 detector; GE, Milwaukee, WI, USA) in the helical mode, with the slice thickness set to 1.25 mm, and the spacing between slices set to 2.5 mm. The images were reviewed by an experienced orthopedic surgeon using Synapse software (Fujifilm Medical Co. Ltd., Tokyo, Japan), and CT images including the GMd were selected (Fig 1a). The number of images containing the GMd was different for each participant according to their physique. All images had dimensions of 512 × 512 pixels.

An overview of the automatic segmentation system proposed in this study is presented in Fig 1.

### Preparation of processing dataset

#### Automatic creation of guide images

**Fig 2:**
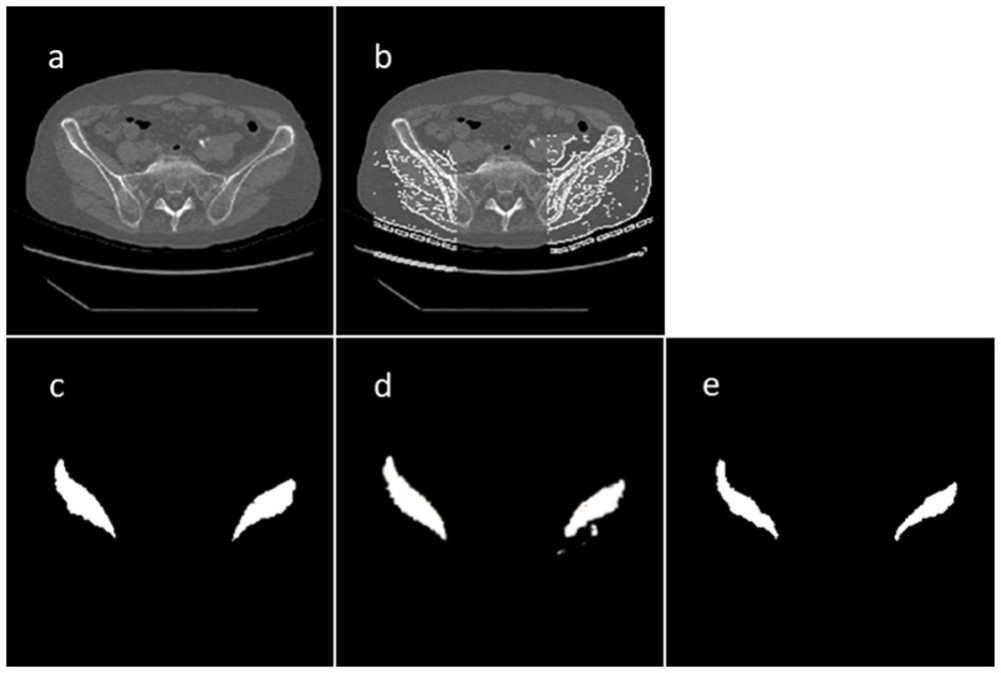
CT and segmentation images: (a) original CT image, (b) guide image using Canny edge detector, (c) manual segmentation image of GMd contours, (d) Auto-segmentation image obtained using learning model with beta1 = 0.9, L1_weight = 1000, and 100 epochs, and (e) manually segmented image prepared by another operator.

The edges of the GMd in the 691 CT images selected for the study were detected using a Canny edge detector [15]. The Open Computer Vision (OpenCV) image analysis library (Version 4.1.1) was used for image processing. The parameters were adjusted and optimized to detect the most effective edge (cv2.THRESH_BINARY: threshold = 140, max value = 200, cv2.Canny: threshold1 = 55, threshold2 = 45). The detected images were used as guide images for manual contour extraction after resizing all images to 217 × 217 pixels according to the format of the original deep learning algorithm described in Fig 2b.

#### Manual segmentation

Based on the edge in the guide image obtained in the previous step, the discontinuity line was complemented, and unnecessary edges were manually deleted; the contours of the GMd on both sides were then extracted (Fig 2c). All extractions were performed by an experienced independent orthopedic surgeon.

The extracted images were used as output images, and they were combined with the original CT images as input images to create 691 image pairs.

#### Split dataset

The data were divided into training, model validation, and test datasets. The data from participants 1 to 5, participant 6, and participant 7 were assigned to the training, model validation, and test datasets, respectively (Table 1).

#### Data augmentation

Research on general image processing using a convolutional neural network requires a large amount of data. Because real data are insufficient, data augmentation methods are generally employed; image augmentation methods are used to create artificial variations in existing images to expand an image dataset [16]. In this study, image pairs in the training and model validation datasets were amplified by a factor of 10 to 6050 sets by applying different combinational transformation techniques including rotating the existing image by 1° or 2° and flipping the existing image horizontally. As the image data were scanned from the upper left pixel toward the lower right, the images created from the existing images in this manner were recognized as new and different images.

#### Manual segmentation

For comparison between the similarity of the output images obtained by automatic segmentation and manual segmentation performed by another operator, the operator independently extracted the GMd contours manually for 86 guide images in the test dataset (Fig 2e). This operator was an experienced orthopedic surgeon.

### Deep learning

In this study, the deep learning model was trained using 5,250 training-data (image) pairs. The model was based on "pix2pix"—the image-generation algorithm proposed by Isola et al. [17]. It represents the original formulation of a conditional GAN for image-to-image translation. In this study, we used its TensorFlow implementation (available at https://github.com/affinelayer/pix2pix-tensorflow). The GAN setup comprises two agents. The first corresponds to a model (discriminator) trained to identify whether an image is real or fake. The second agent is a generator that learns to create realistic new data to convince the discriminator that the samples it generates are from the dataset [18].

The pix2pix generator is an encoder–decoder model that embeds an input image into a low-dimensional vector and restores it to the expected output. This generator uses a structure called U-Net with a skip connection, which directly connects the same layer between the encoder and decoder. By providing the skip connection, the features of the intermediate layers can be propagated directly, and the details can be restored faithfully. The pix2pix discriminator uses the L1 loss function per pixel between the generated and training images to learn the entire image (low-frequency component) and then captures the details of the image (high-frequency component) using a convolutional “PatchGAN” classifier, which only penalizes the structure at the scale of image patches [17]. As PatchGAN divides the entire image into patches and assesses each patch area to identify real/fake images, the features of the high-frequency components can be captured well. The L1 loss function and PatchGAN complement each other to improve the accuracy.

We performed a grid search of the combinations of beta1 and L1_weight using model validation datasets to tune the hyperparameters. Beta1 is the exponential decay rate for the first moment estimates, relative to the Adam optimizer. L1_weight is the weight on the L1 term for the generator gradient. The grids of beta1 values in [0.1, 0.5, 0.9] and L1_weight values in [10, 100, 1000] were explored. For the top three combinations of beta1 and L1_weight, training was continued for up to 100 epochs to determine the optimal number of training epochs.

### Quantitative validation

The OpenCV Version 4.1.1 library was used for the quantitative evaluation. The similarity of the segmented regions was verified using the following three methods.

#### Overlap measure

The dice similarity coefficient (DSC) was intended for application to discrete data [19]. It is commonly used in image segmentation, particularly for comparing the outputs of algorithms against reference masks in medical applications.

Given two sets, A and B, the DSC is defined as

$$\text{DSC}=\frac{2|A\cap B|}{\left|A\right|+\left|B\right|}.$$

The DSC is the quotient of the similarity and ranges between zero and one. In this study, the DSC was calculated using the white pixel count of a binary image.

#### Volume measure

The volume similarity (VS) is defined as

$$VS=\frac{2*(|v_{A}-v_{B}|)}{v_{A}+v_{B}},$$

where *v*_***A***_ and *v*_***B***_ represent the areas of A and B, respectively [20].

#### Shape similarity measure

The seven Hu moment invariants combined with the normalized central moments up to the third order do not change when the image is translated, rotated, or scaled [21]. The shape similarity can be obtained with a MatchShapes (MS) function that represents the difference in shape obtained by calculation based on Hu moment invariants. MS is defined as

$$I1\left(A,B\right)=\sum_{i=1\ldots 7}\left|\frac{1}{m_{i}^{A}}-\frac{1}{m_{i}^{B}}\right|$$

$$m_{i}^{A}=\text{sin}(H_{i}^{A})∙\text{log}H_{i}^{A}$$

$$m_{i}^{B}=\text{sin}\left(H_{i}^{B}\right)\cdot \text{log}H_{i}^{B},$$

where $H_{i}^{A}\;and\;H_{i}^{B}$ represent the Hu moment invariants of A and B, respectively.

The contour line of the segmentation area was detected by applying the FindContours function of OpenCV [22]; the cv2.matchshapes_I1 function (MatchShapes), which is an OpenCV intrinsic function, was used for the calculations. The MS function performs matching using shape information and returns a number representing the difference between the shapes calculated based on the moment values. The smaller is the number, the more similar are the two shapes.

The GMd-extracted images automatically generated by the trained model and output images were compared, and the DSC was used to search for optimal hyperparameters using the model validation dataset. The DSC, VS, and MS were used to compare the segmentation results of manual segmentation and automatic segmentation (hereafter, auto-segmentation) in the test dataset.

### Statistical analysis

The normality of the data distribution was confirmed using the skewness and kurtosis of the univariate distribution. Then, a paired *t* test was performed. We set 10% of the average similarity score of the manual segmentation by another operator as an acceptable noninferiority limit value and performed the noninferiority test for auto-segmentation using the similarity score of the manual segmentation as a control.

## Results

The results of the grid search for the combinations of beta1 and L1_weight using the model validation datasets are summarized in Table 2. The top three combinations of beta1 and L1_weight extended the training to 100 epochs. The DSC results are described in Table 3. The average DSC of the output images and auto-segmentation reached a peak of 0.728 in [beta1 = 0.9, L1_weight = 1000] at 100 epochs. Based on this result, the similarity with the output images was examined using this learning model.

**Table 2:** Results of grid search for the combinations of beta1 and L1_weight in the model validation datasets.

DSC of model trained on 20 epochs	Beta1 = 0.1	Beta1 = 0.5	Beta1 = 0.9
**L1_weight = 10**	0.008	0.614	0.513
**L1_weight = 100**	0.651	0.625	0.62
**L1_weight = 1000**	0.704	0.707	0.702

DSC: dice similarity coefficient.

**Table 3:** Results of grid search for beta1 and L1_weight combinations as well as training epochs in the model validation datasets.

Epochs	20	30	40	60	80	100
**beta1 = 0.5; L1_weight = 1000**	0.707	0.715	0.694	0.698	0.683	0.707
**beta1 = 0.1; L1_weight = 1000**	0.704	0.653	0.708	0.727	0.668	0.682
**beta1 = 0.9; L1_weight = 1000**	0.702	0.684	0.672	0.694	0.708	0.728

**Fig 3:**
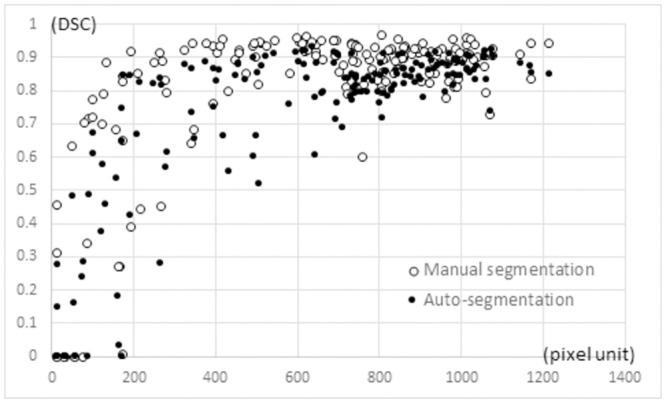
Scatter plot of CSA (unit: Pixels) and DSC in the model validation datasets.

**Fig 4:**
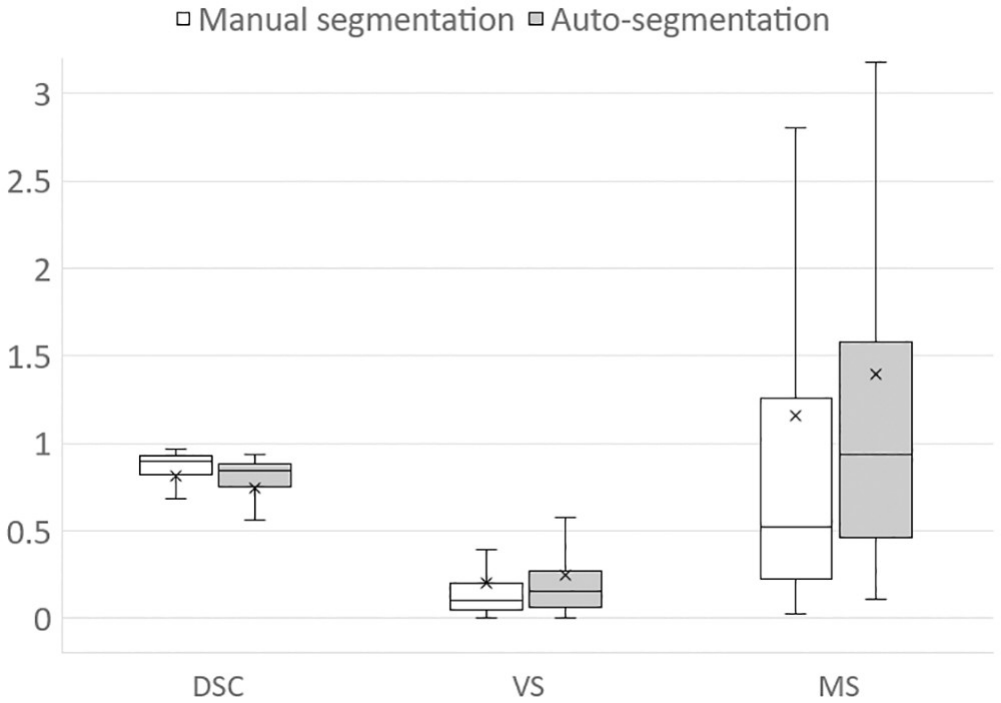
Comparison of segmentation similarity between two segmentation methods.

The average DSC for auto-segmentation was 0.748, and the average for manual segmentation was 0.812, with a significant difference (p < 0.0001). The scatter diagram showed a difference in the DSC tendency depending on the CSA of the GMd (Fig 3). The average VSs for auto-segmentation and manual segmentation were 0.247 and 0.203, respectively, with no significant difference (p = 0.069). Furthermore, the average MSs for auto-segmentation and manual segmentation were 1.394 and 1.156, respectively, with no significant difference (p = 0.308). The results of the segmentations obtained using the three methods are compared in Fig 4. The mean volume of GMd obtained by auto-segmentation on both sides was 282.9 cm^3^. For operator1 and operator2, the mean volume of GMd obtained by manual segmentation was 261.6 cm^3^ and 246.2 cm^3^, respectively.

**Fig 5:**
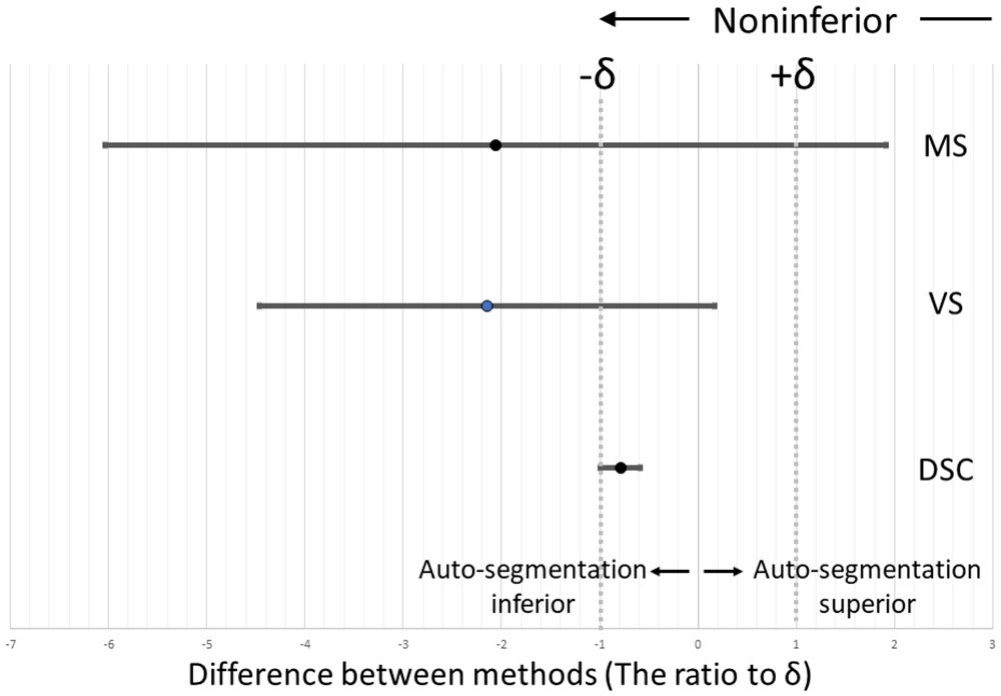
Noninferiority test comparing automatic and manual segmentation techniques using the 95% confidence interval of difference between two groups: δ: Equivalence margin (10% of average similarity score of manual segmentation performed by another operator).

The noninferiority test showed that the lower 95% confidence interval values for the DSC surpassed the lower limit of the range of equivalence margins, verifying the noninferiority of the auto-segmentation outcomes (Fig 5).

## Discussion and conclusions

Several muscle-analysis studies have been performed using magnetic resonance imaging [23, 24], which depicts soft tissues in better contrast than CT scans. Because CT is often used to manage hip OA, including surgical treatment, muscle volume evaluation using CT is considered more convenient. However, there is high inter-operator variability in manual muscle segmentation because the muscle boundary is difficult to identify (Fig 2c and 2e).

In recent years, deep learning and generative models have been widely adopted in musculoskeletal radiology [6, 7]. Deep learning involves a multi-layered model constructed using a neural network that imitates human neural circuits and performs highly accurate inferences; great progress was made in this area in the 2010s. Since then, deep learning-based research and development have become active, and drastic improvements have been achieved in image and speech recognition. Currently, deep learning is used in several fields, including systems for playing intellectual games, such as chess and Shogi; image-recognition systems (computer vision) for identifying objects and people in images and videos; speech-recognition systems that listen to human speech and understand instructions; advanced and autonomous control systems for machines, such as robots and automobiles (autonomous driving); natural language processing systems, such as automatic summarization and question answering systems; and advanced and natural machine-translation systems [25].

In this study, we applied a deep generative model using GANs and attempted automatic GMd segmentation using the CT images of hip OA patients. Deep generative models, which consist of deep neural networks for generative models focusing on the data generation process, can learn high-dimensional and large-scale data; furthermore, they can generate high-quality images because of the expressive power of the network, which has attracted considerable attention. GANs are generative models; by extracting and learning the features of data, nonexistent data can be generated or transformed along with the existing data features. Additionally, GANs comprise two neural networks, and these networks improve each other’s accuracy by using multiple models called the generator and discriminator. This characteristic method generates highly accurate data that are difficult for humans to distinguish as the learning step (epoch) progresses. As the performance of deep learning plateaued in the present study, we did not increase the number of epochs further.

In this study, the DSC of auto-segmentation was extremely low in slices with small CSAs of the GMd (Fig 3). In the slice at the distal GMd, the periphery was surrounded by the gluteus minimus, tensor fasciae latae, and gluteus maximus, and it was difficult to identify the boundaries between muscles with similar CT values. Therefore, manual segmentation had to be performed with reference to the position and morphology of the GMd in the preceding and subsequent slices. By contrast, in the slice containing the proximal GMd, the GMd was tangent to the ilium, which facilitated manual segmentation, but it could not be generated by auto-segmentation. This difference may have affected the overall performance.

In the noninferiority test, which was performed considering the similarity performance realized via manual segmentation control, VS and MS did not demonstrate the auto-segmentation noninferiority (Fig 5). However, considering that manual segmentation requires a processing time of approximately 5 min per slice and more than 6 h per case, these results are worth considering for clinical use.

During the abovementioned noninferiority test, the DSC values corresponding to the lower 95% confidence interval surpassed the lower limit of the equivalence-margin range. This confirms the noninferiority of the auto-segmentation outcomes. Contrastingly, VS did not demonstrate the auto-segmentation noninferiority. Therefore, the auto-segmentation of GMd using deep conditional GANs is not inferior to manual segmentation in terms of the segmented-area commonality. Automation reduces the effort and time required for muscle segmentation and therefore facilitates an accurate assessment of the cubic muscle volume, which makes it usable in medical practice. Although clinical validation of severe hip OA is yet to be undertaken, the proposed method demonstrates the potential for use to that end without requiring special reconstruction techniques.

## Supporting information

S1 Data (XLSX)
